# Valvular Hemolysis Masquerading as Prosthetic Valve Stenosis

**DOI:** 10.7759/cureus.1143

**Published:** 2017-04-08

**Authors:** Pooja Sethi, Ghulam Murtaza, Zia Rahman, Syed Zaidi, Thomas Helton, Timir Paul

**Affiliations:** 1 Cardiology, East Tennessee State University; 2 Internal Medicine, East Tennessee State University; 3 Internal Medicine, James H. Quillen Veteran Affairs Medical Center

**Keywords:** high output states, prosthetic valves, gradient

## Abstract

The evaluation of prosthetic valves can provide a unique challenge, and a thoughtful approach is required. High output states like anemia should be kept in the differential when evaluating elevated gradients across prosthetic valves. We present the case of a 69-year-old man with a Starr-Edwards prosthetic aortic valve who presented with symptoms of congestive heart failure and high transvalvular pressure gradients. These symptoms indicate a potential prosthetic valve stenosis. His laboratory evaluation results were consistent with valve-related hemolysis. Resolving his anemia led to a resolution of the symptoms and lowered the pressure gradient on follow-up.

## Introduction

Valve-related hemolysis is one of the potential complications related to prosthetic heart valves. Hemolysis usually results from the structural deterioration of the valve or a paravalvular leak. Severe hemolytic anemia can lead to a high-flow state resulting in high-pressure gradients across the valve. This increased gradient may masquerade as valve stenosis. In addition to valve dysfunction, hemolysis should be considered among the important differentials of elevated transvalvular gradients. These cases can be easily managed with supportive therapy without the need for valve replacement.

## Case presentation

**Figure 1:**
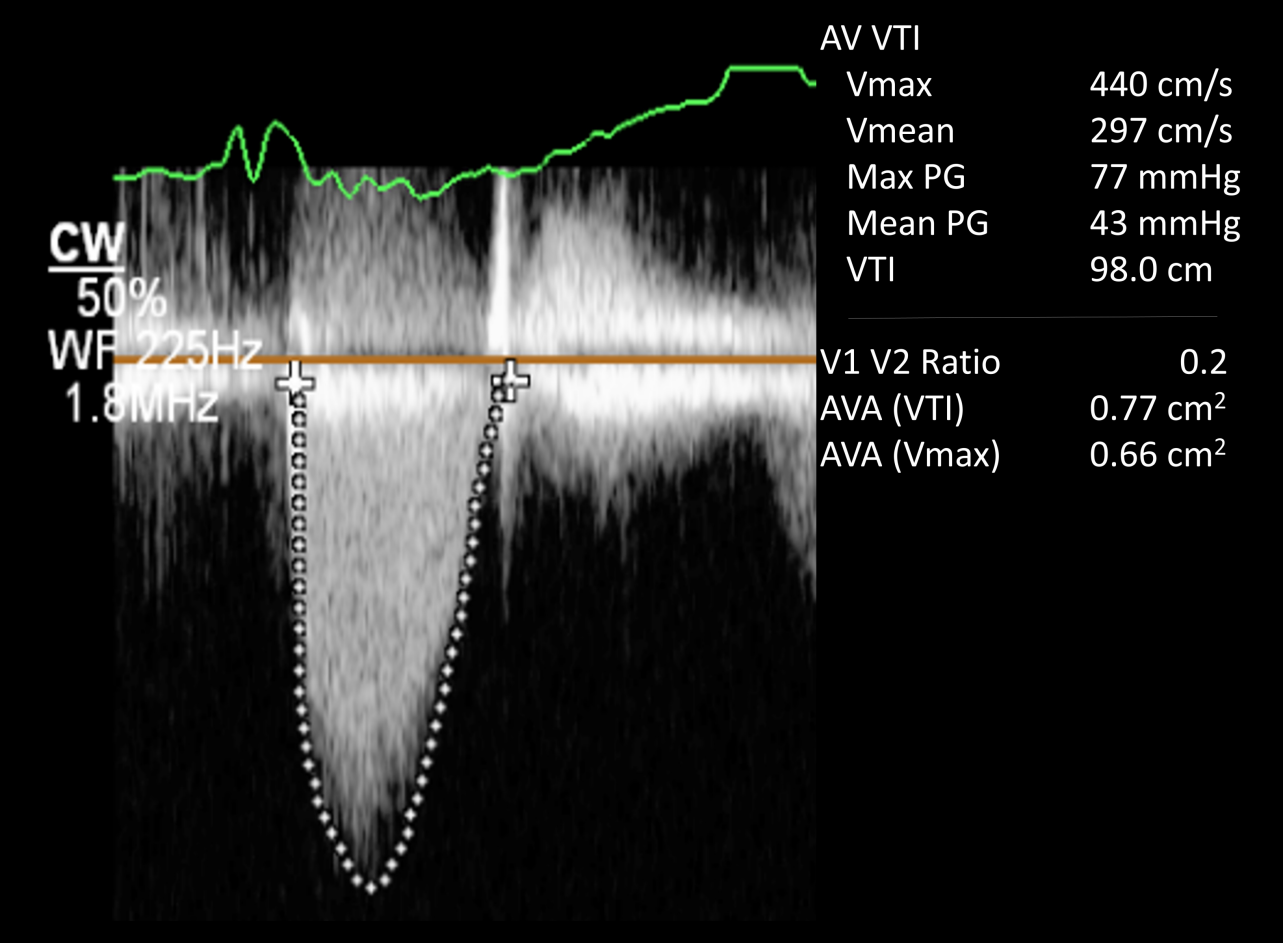
Doppler echocardiogram showing elevated peak and mean gradients across the aortic valve at 77 mm Hg and 43 mm Hg respectively

**Figure 2:**
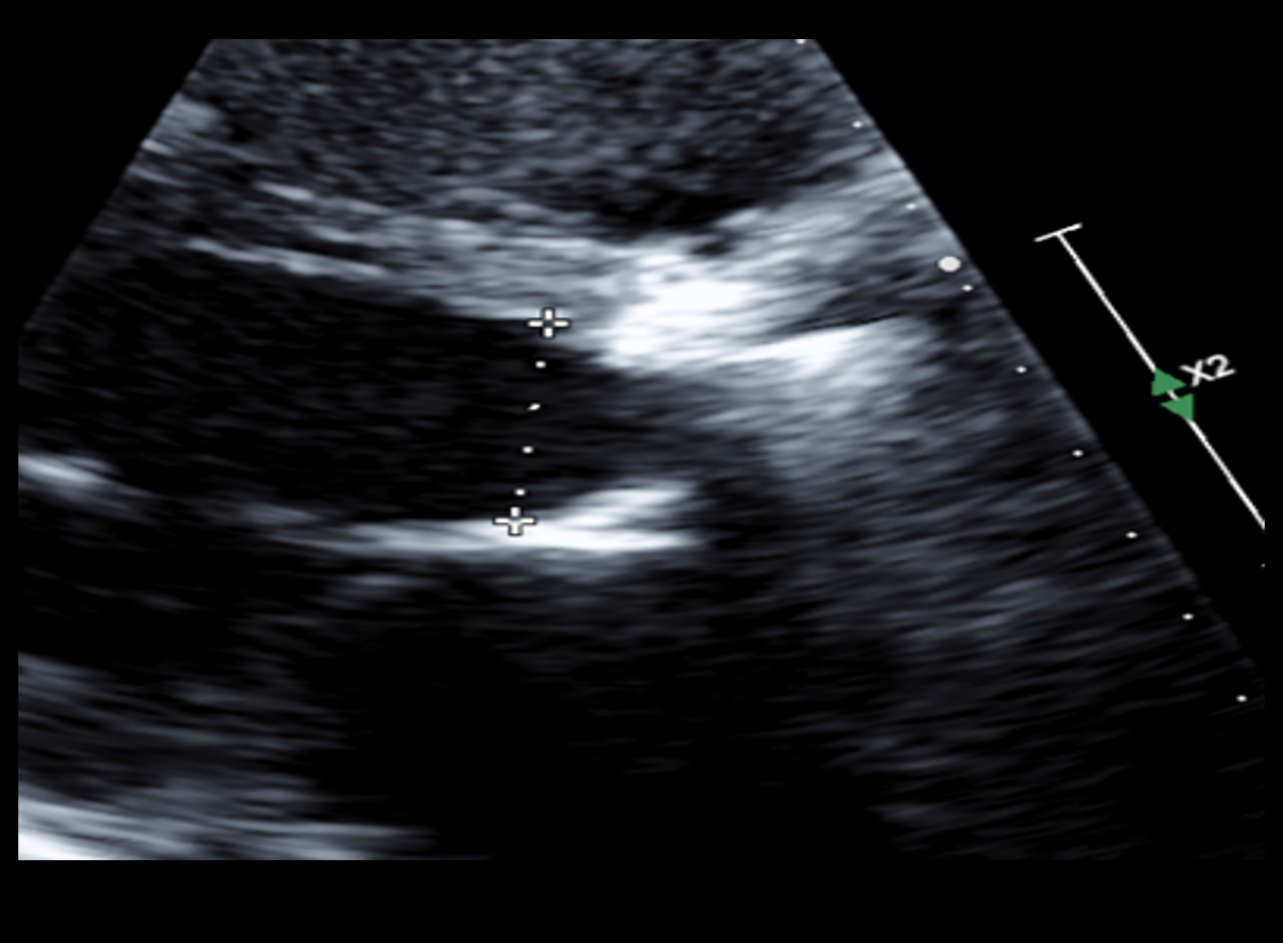
Echocardiogram showing Ball and Cage, and Starr Edwards valve

**Figure 3:**
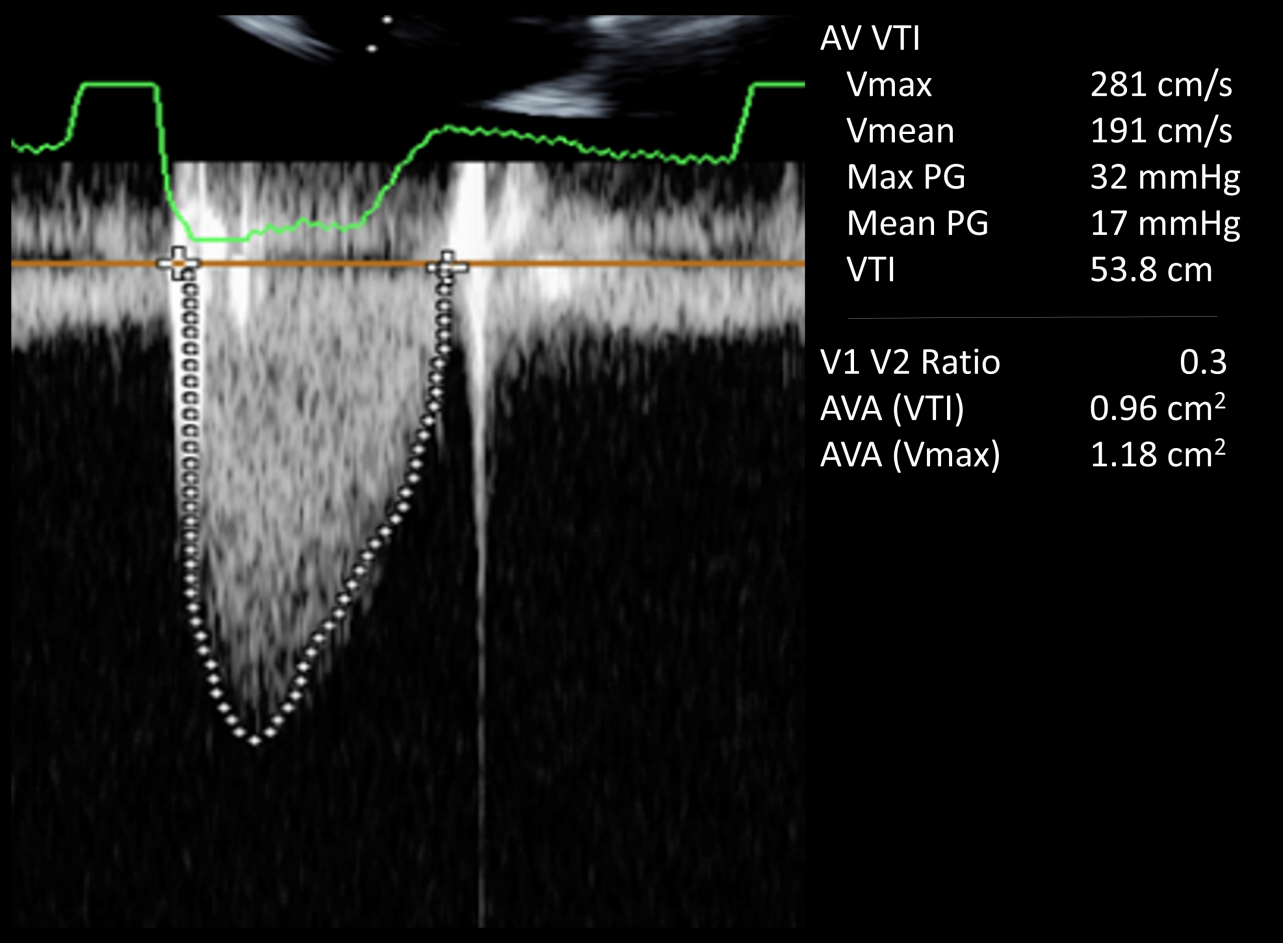
Doppler echocardiogram showing improvement in peak and mean gradients at 32 mm Hg and 17 mm Hg respectively with treatment of anemia

A 69-year-old man, with a medical history significant for severe aortic stenosis after a Starr-Edwards prosthetic valve replacement in 1968, presented with worsening dyspnea on exertion, pedal edema, and progressive weight gain of 15 lb over the course of the preceding six to eight weeks. On physical examination, his blood pressure was 110/80 mmHg, pulse was 65 beats/minute, respiratory rate was 18 breaths/minute, and his oxygen saturation was 95% on 2L of oxygen. Other pertinent findings included mild inspiratory crackles at lung bases and 1+ bilateral lower extremity edema. The complete blood count showed a hemoglobin level at 8 g/dL with reduced mean corpuscular volume. His baseline hemoglobin level was 14 g/dL three months prior to presentation. His white cell count, platelet count, and serum chemistries were all normal. Iron studies showed decreased serum ferritin levels. His electrocardiogram (EKG) evaluation was unremarkable and a chest x-ray revealed pulmonary venous congestion. He was started on intravenous (IV) furosemide. A transthoracic echocardiogram (TTE) showed a normal ejection fraction; more importantly, it demonstrated a dramatic increase in aortic valve gradients (which had been normal on the previous TTEs). His peak and mean gradient were 77 and 43 mmHg respectively with a dimensionless velocity index of 0.24 (Figure 1). The aortic valve appeared normal in structure and function with a calculated valve area of 0.82 cm^2^ (Figure 2). Given the patient’s age and the presence of iron deficiency anemia, the gastroenterology department was consulted and a workup for upper and lower gastrointestinal tract that included capsule endoscopy was unrevealing for any source of bleeding. A hemolysis panel was obtained that revealed low haptoglobin levels ( < 10 mg/dL) and elevated lactate dehydrogenase (LDH) levels ( > 1000 U/L) consistent with intravascular hemolysis. He received three units of blood. His symptoms improved considerably and he was discharged on iron supplementation therapy. The patient was evaluated at a six-week follow-up office visit. The follow-up TTE demonstrated gradients at baseline with a peak and mean gradient of 32 and 17 mmHg respectively, which is normal for a Starr-Edwards valve (Figure 3).

## Discussion

Intravascular hemolysis is one of the major complications associated with prosthetic heart valves. in 1975, Kloster, et al. reported the incidence of severe hemolytic anemia in patients with prosthetic heart valves as five percent to fifteen percent[1]. With current advances in surgical techniques and newer generation prosthetic heart valves, the incidence of severe hemolysis leading to clinically significant anemia has decreased to less than one percent [2]. Skoularigis, et al. evaluated 250 patients with different types of mechanical prostheses with normal mechanical valve function and found no incidences of decompensated anemia. However, mild compensated hemolysis was quite frequent with a 51.2% incidence in patients with St. Jude Medical® Mechanical Heart Valve (St. Jude Medical Inc., St. Paul, MN) and 17.8% in patients with Medtronic-Hall valves (Medtronic, Inc., Minneapolis, MN) [3].

Mechanical trauma to red blood cells from turbulent blood flow is the main mechanism for hemolysis. This turbulence creates shear stress on red blood cells resulting in their destruction [4]. In addition to turbulence, regurgitation of blood during valve closure can induce shear stress that leads to chronic low-grade hemolysis [5]. The higher rate of hemolysis in older-generation prosthetic valves was due to structural deterioration of the valve over time. Newer-generation prosthetic valves are more durable and structural deterioration is extremely rare. The most common mechanism explaining hemolysis with these prostheses is periprosthetic leakage [6].

The clinical presentation of a patient with hemolytic anemia related to prosthetic valves is variable and depends on the duration and severity of the anemia. Usually, hemolysis is subclinical and diagnosed via routine laboratory evaluations with findings such as a mild drop in hematocrit and a mild elevation of LDH. Clinical findings usually include signs of anemia, jaundice, dark urine, new onset murmur or change in the characteristics of the murmur [7]. In severe cases, a patient may present with signs of fluid overload from high-output heart failure.

The laboratory evaluation of these patient involves the presence of anemia, elevated LDH, low haptoglobin, elevated reticulocyte count, and a peripheral blood smear showing fragmented red blood cells called schistocytes. One important concept to note is that these patients may not develop anemia unless their bone marrow fails to compensate for the shortened lifespan of the red blood cells. In such cases, other accompanying laboratory markers might be helpful for the diagnosis of hemolysis.

An echocardiogram, to evaluate prosthetic valve functions, becomes useful in the context of an appropriate clinical scenario and suggestive laboratory findings. A Doppler echocardiogram would allow for measuring the direction and velocity of blood flow. It also helps in calculating pressure gradients across prosthetic valves [8]. TTE images are especially useful in patients with an aortic prosthesis.

The multiple etiologies of elevated pressure gradients across aortic valve prostheses are summarized in Table 1 [9]. High-flow states such as high-output heart failure from valve-related hemolysis would result in increased transvalvular pressure gradients leading to a false impression of severe aortic stenosis. Cardiologists should bear this concept in mind during routine practice. These patients respond to blood transfusion and iron supplementation which leads to the normalization of gradients over several weeks to months. Our patient had an older generation Starr-Edwards valve model. This valve was cloth-covered at the time of implantation. A tear in the cloth over time can lead to valve-related hemolysis several years after its implantation [10]. In our patient, the mechanical aortic prosthesis led to progressive hemolysis and ultimately to a high-output heart failure. His baseline pressure gradients were normal. The differential for the sudden elevation in prosthetic aortic valve gradient included valve thrombosis, pannus formation, patient-prosthetic mismatch, and high-output states. His anemia was likely secondary to valve-related hemolysis which led to elevated gradients. By correcting his anemia, the gradients improved. He has been stable on long-term iron supplementation with improved anemia and clinical symptoms at his three-month follow-up evaluation.

**Table 1:** Etiologies of elevated pressure gradients across aortic valve prostheses Abbreviations: MR, mitral regurgitation; LV, left ventricle.

No LV-Outflow Obstructions	LV-Outflow Obstruction
Measurement error	Obstruction at the aortic valve
Signal contamination or confusion with MR	Prosthesis dysfunction
Correction for “cosine theta”	Bioprosthesis calcification
Over-tracing spectral Doppler envelope	Thrombus or vegetation
High-flow state	Pannus overgrowth
Fever, anemia, hyperthyroidism, anxiety, regurgitation	Patient-prosthesis mismatch
Pressure recovery	Subvalvular or supravalvular obstructions

## Conclusions

When evaluating prosthetic valves, alternative causes of elevated transvalvular gradients should be considered if there is a sudden increase in transvalvular gradients in an otherwise normally functioning prosthetic valve. Anemia with high-output heart failure due to valve-related hemolysis can lead to spuriously elevated transvalvular gradient, which would be a differential in assessing prosthetic valve stenosis. 
